# Not All Mice Are Equal: Welfare Implications of Behavioural Habituation Profiles in Four 129 Mouse Substrains

**DOI:** 10.1371/journal.pone.0042544

**Published:** 2012-08-03

**Authors:** Hetty Boleij, Amber R. Salomons, Mariska van Sprundel, Saskia S. Arndt, Frauke Ohl

**Affiliations:** 1 Faculty of Veterinary Medicine, Department of Animals in Science and Society, Division of Animal Welfare and Laboratory Animal Science, Utrecht University, Utrecht, The Netherlands; 2 Rudolf Magnus Institute of Neuroscience, Utrecht, The Netherlands; Université Pierre et Marie Curie, France

## Abstract

Safeguarding the welfare of animals is an important aim when defining housing and management standards in animal based, experimental research. While such standards are usually defined per animal species, it is known that considerable differences between laboratory mouse strains exist, for example with regard to their emotional traits. Following earlier experiments, in which we found that 129P3 mice show a lack of habituation of anxiety related behaviour after repeated exposure to an initially novel environment (non-adaptive profile), we here investigated four other 129 inbred mouse substrains (129S2/SvPas, 129S2/SvHsd (exp 1); 129P2 and 129X1 (exp 2)) on habituation of anxiety related behaviour. Male mice of each strain were repeatedly placed in the modified hole board test, measuring anxiety-related behaviour, exploratory and locomotor behaviour. The results reveal that all four substrains show a lack of habituation behaviour throughout the period of testing. Although not in all of the substrains a possible confounding effect of general activity can be excluded, our findings suggest that the genetic background of the 129 substrains may increase their vulnerability to cope with environmental challenges, such as exposure to novelty. This vulnerability might negatively affect the welfare of these mice under standard laboratory conditions when compared with other strains. Based on our findings we suggest to consider (sub)strain-specific guidelines and protocols, taking the (subs)train-specific adaptive capabilities into account.

## Introduction

Animal welfare is an important issue to consider in laboratory animal research and management, from both an ethical point of view and for generating reliable experimental results. Legal frameworks and more detailed guidelines are an important instrument used to safeguard the welfare of experimental animals. Currently the protection of animal welfare is primarily directed on the absence of negative factors such as illness, distress hunger, pain, anxiety and fear (five freedoms [1]) and the promotion of natural behaviour. However, recently the importance of the presence of positive emotions [2]–[5] as well as the relevance of the animals' adaptive capacities [6]–[8] have been discussed, and it has been suggested to consider the animals' freedom to adequately react to prevailing environmental circumstances as an indicator for the individuals' welfare [4].

It is clear that safeguarding animal welfare demands the definition of criteria for both measurable animal based parameters (such as behavioural and/or physiological parameters) and environmental parameters (such as animal housing and management measures, see for example the Welfare Quality project [9]). These criteria have to take animal-specific characteristics into account and today's guidelines usually define minimal welfare demands per animal species. However, one may wonder whether indeed ‘a mouse is a mouse’ [10] when aiming at safeguarding the welfare of the variety of existing laboratory mouse strains, and especially with respect to appreciating the expression of their ‘natural’ behaviour. Selective breeding programmes in laboratory animals are often focused on physiological characteristics, but selection may have an (unintended) impact on other characteristics, such as emotional traits and related behavioural expressions (see for example [8], [11]–[15]). Such characteristics then may result in reduced adaptive capacities, which in turn can compromise biological functioning and thus may finally impair welfare in (selectively bred) animals [6], [8].

The adaptive capacity of an animal depends on the genetic background as well as on environmental and epigenetic factors [16], [17]. Strain comparisons in small rodents reveal that the same environmental stimulation can elicit varying behavioural responses (see for example the effects of environmental enrichment and chronic stress on behaviour of different mouse strains [18]–[20]). Moreover, gene-environment interactions may influence habituation processes and may affect adaptive capacities, as can be seen in inbred or selectively bred rodent strains. For example, so-called LAB-rats (Low Anxiety Behaviour) and C57BL/6 mice initially show non-anxious behaviour in a novel environment and reveal no further habituation during repeated exposure, while HAB-rats (High Anxiety Behaviour) as well as DBA/2 mice show initially high anxious behaviour, but reveal rapid habituation during repeated exposure [13], [21].

Anxiety is a highly conserved adaptive emotion that occurs in situations of potential danger or threat and is one of the so-called “negative” emotional states that at least all vertebrates are supposed to be able to experience [22]. For example exposure to a novel situation or environment induces a state of anxiety due to the uncertainty of this environment. By exploring a novel environment, it becomes more familiar and anxiety decreases. The process of waning of a certain behavioural response over time is also described as behavioural habituation [23]. Hence the ability of an animal to habituate is a reflection of the capacity of this animal to adapt to the situation and *vice versa* [6].

In previous studies we found that the 129P3/J substrain shows a lack of habituation during repeated exposure to an initially novel environment [24]. In comparison with the initially highly anxious BALB/c mouse, the 129P3/J substrain shows low initial avoidance behaviour but over time this behaviour increases [24], indicating a fundamental inability to adapt [24]. Such differences in adapting to novelty implicate that different mouse-strains may respond very differently to standardized housing-conditions that are defined for mice as a species in general [25], which actually brings up the question whether one and the same guideline regarding housing conditions may be feasible to safeguard welfare in all laboratory mouse strains, or, as suggested earlier, that it may be necessary to define strain-specific guidelines [6].

In extension of earlier strain-comparisons, we here investigated four 129 *sub*strains on their habituation behaviour to evaluate whether structural differences in adaptive capacities have to be taken into account even at a more differential level than the *strain*-level. This is a relevant question since other studies have demonstrated that there is substantial genetic and phenotypic variation between the 129 substrains [26]–[30]. In two independent experiments we repeatedly exposed mice of the 129S2/SvPasCrl (129S2Pas) and 129S2/SvOlaHsd (129S2Hsd) (experiment 1) and mice of the 129X1/J (129X1) and 129P2/OlaHsd (129P2) (experiment 2) to an initially novel testing environment. These specific substrains were chosen because of their frequent use in laboratory research, their relatedness [26], and because of the previous findings (see above) in one of the existing 129P3 substrains. On basis of our results we hope to draw conclusions on the general adaptive capacities of the 129 strain and what this might mean for management protocols for safeguarding their welfare under standard laboratory housing conditions.

## Materials and Methods

### Ethical Note

The experimental protocols (DEC numbers 2007.I.01.007 and 2009.I.10.079) were approved by the Animal Experiments Committee of the Academic Biomedical Centre Utrecht, The Netherlands. The Animal Experiments Committee based its decision on the EC Directive 86/609/EEC (Directive for the Protection of Vertebrate Animals used for Experimental and other Scientific Purposes). Furthermore, all animal experiments followed the ‘Principles of Laboratory Animal Care’ and refer to the Guidelines for the Care and Use of Mammals in Neuroscience and Behavioural Research (National Research Council 2003).

### Animals and Housing

The behavioural experiments were performed at two different locations. In both experiments similar housing conditions and experimental procedures were applied. The dark period started at 6.00 and lasted until 18.00 h (reversed light/dark cycle) and radio music was turned on as background noise during the whole experimental period. During the first two weeks after arrival (habituation period) the animals were handled for ∼3 minutes per mouse three times a week (between 9 and 11 AM) by the experimenter who also did behavioural testing. All testing took place in the animals housing room and equipment was installed before the animals arrived.

#### Experiment 1

This experiment took place at the Central Laboratory Animal Research Facility of Utrecht University (location Paviljoen) with 8 male 129S2/SvPasCrl (129Pas, Charles River, Germany) and 8 male 129S2/SvHsd (129Hsd, Harlan, The Netherlands) mice. The animals were 7–8 weeks old at arrival and housed individually in Eurostandard Type II cages (size: 365×207×140 mm, floor area 530 cm^2^; Techniplast, Milan, Italy) with standard bedding material (Aspen chips; Abedd-Dominik Mayr KEG, Köflach, Austria), a tissue (KLEENEX® Facial Tissue, Kimberly-Clark Professional BV, Ede, The Netherlands) a cardboard shelter and some cardboard shredding (Envirodri®, Technilab-BMI BV, Someren, The Netherlands) as cage enrichment. The mice were kept in the test room for 17 days under constant laboratory conditions for acclimatisation to the experimental room with water and food (CRM, Expanded, Special Diets Services Witham, England) available ad libitum. Relative humidity was kept at a constant level of approximately 50% (±5%), room temperature was sustained at 22°C±2 and ventilation rate was 15–20 air changes per hour.

#### Experiment 2

The second experiment took place at the Central Laboratory Animal Research Facility of Utrecht University (location GDL) with 8 male 129P2/J (129P2, Harlan Europe, UK) and 8 male 129X1 (129X, Jackson Laboratory, USA) mice. The mice were 7–8 weeks old at arrival and housed individually in Eurostandard Type II cages (size: 365×207×140 mm, floor area 530 cm^2^; Techniplast, Milan, Italy) with standard bedding material, a tissue, and a cardboard shelter as cage enrichment. The mice were kept in the test room for 17 days (129X1) and 23 days (129P2) under constant laboratory conditions for acclimatisation to the experimental room with water and food (CRM, Expanded, Special Diets Services Witham, England) available ad libitum. Relative humidity was at a constant level of approximately 50%, room temperature was sustained at 22°C±2 and ventilation rate was 15–20 air changes per hour.

### Modified Hole Board (mHB)

The mHB consisted of a an opaque grey PVC box (100×50×50 cm) with a hole board, which was made of the same material as the box, positioned in the middle of the box (60×20×2 cm), thus representing the unprotected area comparable with the centre of an open field. On the board 20 cylinders (15×15 mm) were staggered in three lines. The area around the board was divided by black lines into 10 rectangles (20×15 cm) and 2 squares (20×20 cm), the number of lines crossed were used to get an indication of locomotor activity. The box was illuminated with 1–5 lux (red light), the board was illuminated with a stage light of about 120 lux (white light). For testing, all animals were individually placed in the mHB, always starting from the same corner. Each trial lasted 5 minutes, 4 trials per day over 5 consecutive days (20 trials in total) were performed. For investigation of food intake inhibition, each animal received a piece of almond daily for three days in its home cage before the start of the experiment. The familiar (almond) and unfamiliar food object (dustless precision pellets, 45 mg, Bio-Serv) were also placed in the mHB, always in the same corner, either one positioned at the same distance from the wall.

After each trial, the mHB was carefully cleaned with tap water and a damp towel. All tests were videotaped for raw data storage and behaviour was directly scored by a trained observer using the program Observer 5.0 (Noldus Technology, The Netherlands). The following behavioural parameters were measured and assigned to different behavioural categories according to previous studies [31]; *avoidance behaviour*: the latency until the first board entry, the percentage of time spent on the board and the total number of board entries; *risk assessment*: the number of stretched attend postures and the latency until the first stretched attend; *locomotor activity*: the total number of line crossings, the latency until the first line crossing, the total time spent immobile and the latency until the first immobility event; *general exploration*: the total number of rearings in the box and on the board, the latency until the first rearing in the box and on the board, the total number of hole explorations and the latency until the first hole exploration (a hole was counted as explored when the animal's nose was directed to a hole; direct contact with the hole was not necessary); *directed exploration*: the total number of holes visited, (a hole was counted as visited when the mouse dipped the nose below the rim of the hole) and the latency until the first hole visit; *food intake inhibition*: the latency until the first exploration of the unfamiliar and familiar food object; *arousal or de-arousal*: the percentage of time spent self-grooming, the latency until the first self-grooming event, the total number of self-grooming events and the total number of faecal boli; *escape behaviour*: the total number of jumps.

### Corticosterone

Basal blood samples were collected four days before the start of the experiment (3 PM, around the same time the animals started their last mHB trial) to determine basal corticosterone (CORT) plasma levels. Blood sampling and decapitation took place in a room adjacent to the experimental room (in order not to disturb circadian rhythm of the mice, the intermediate hallway and rooms were under red light conditions in both locations). A small blood sample (50 µL) was collected by tail vein incision, and stored in pre-chilled Microvette tubes (CB300, Sarstedt, Numbrecht, Germany) containing lithium heparin. Two-and-a-half hours after the last trial, animals were decapitated and trunk blood was collected in Minicollect tubes (1 ml Lithium Heparin, Greiner Bio-One GmbH, Kremsmünster, Austria). Plasma CORT levels were measured by radioimmunoassay (RIA) according to the protocol of the supplier with an ImmuChem™ Double Antibody Corticosterone kit for rats and mice (MPI Biochemicals, Amsterdam, The Netherlands).

### Statistical Analysis

Statistical analyses were performed using the software program SPSS 16.0.1 for Windows (SPSS Inc. IL, USA). Continuous data (plasma CORT, latency and relative duration of behavioural parameters) were represented as mean ± standard error of the mean (SEM), and were first investigated for gaussianity using the Kolmogorov-Smirnov test. Homoscedasticity was tested by Levene's test. Some of these parameters revealed a non parametric distribution and were rank transformed. Discrete data on the ordinal scale (total number of behavioural parameters) are represented as median with interquartile range (IQR), and were rank transformed. Behavioural data from the mHB experiments were subsequently analysed using linear mixed model analysis. If a certain behaviour did not occur during the trial latencies were set at 300 s (total trial time). Before analyses, the most appropriate test for each parameter was defined by varying the linear mixed model test with or without random intercept and slope. Based on the value of the 2-log likelihood of the Chi-square distribution, the significantly best test (i.e.) was used for analysis for each specific parameter. This included a linear mixed model analysis with fixed effects of strain, trial x strain interaction and a random intercept or random effects of strain, strain x trial interaction and a random intercept. For linear mixed model analyses a probability value less than 0.05 (two-tailed) was considered as statistically significant. CORT analyses were done using paired (basal/non-basal) or unpaired (strain) Student *t*-tests. The probability value was adjusted for the number of comparisons using Dunn-Šidak correction (α = 1−0.95^1/q^, q is number of comparisons).

## Results

A summary of the results (comparison 1^st^ and 20^th^ trial, to get an indication of the change over time) from the first and second experiment are listed in table S1.

### Avoidance Behaviour

#### Experiment 1: 129S2Pas vs. 129S2Hsd

**Figure 1:**
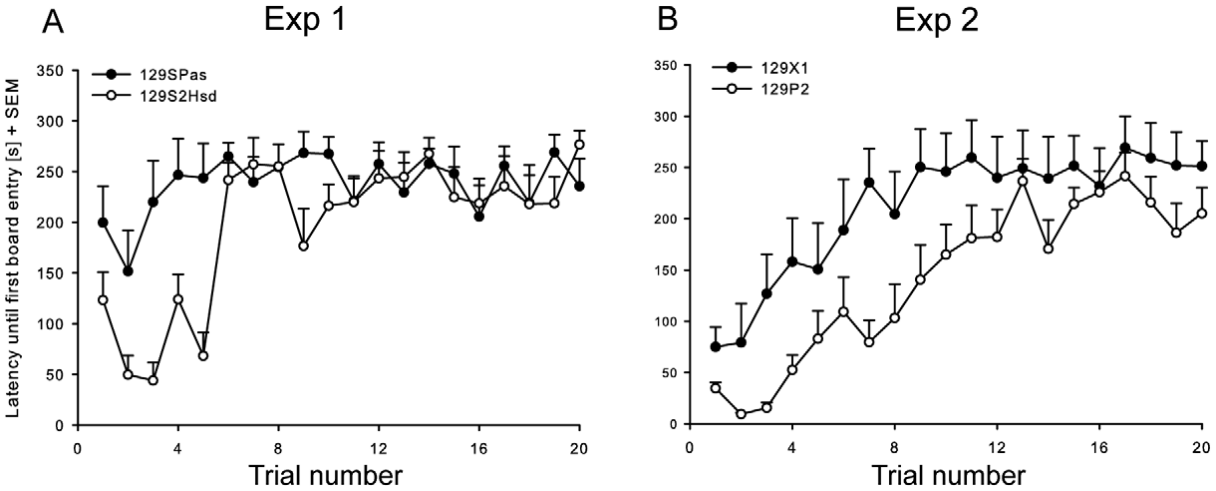
Mean latency from the start of the trial until the first board entry (seconds + SEM). (1A) Experiment 1: 129SPas and 129S2Hsd mice, (1B) experiment 2: 129P2 and 129X1 mice.

**Figure 2:**
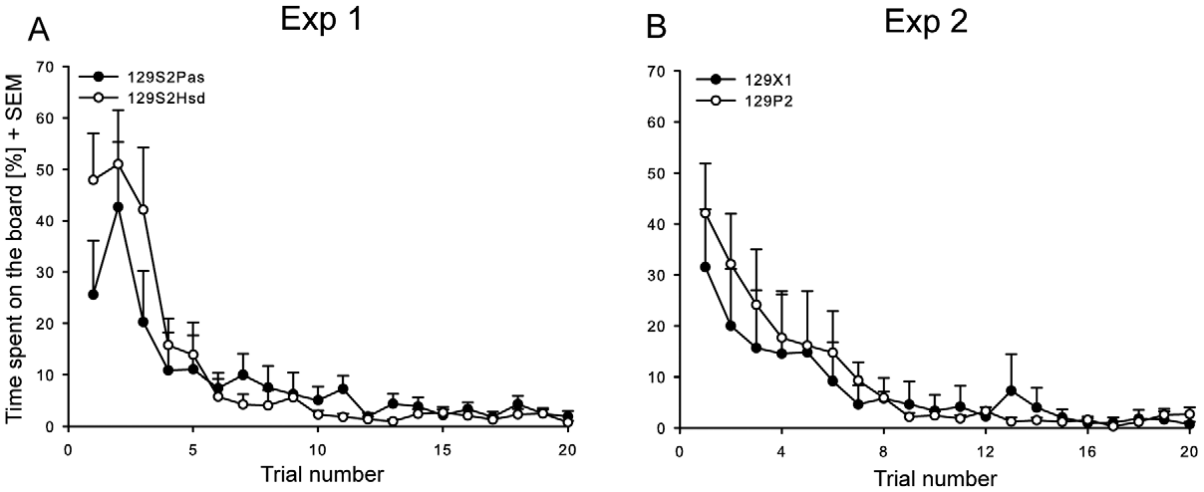
Total percentage of time spent on the board (% + SEM). (2A) Experiment 1: 129S2Pas and 129S2Hsd mice, (2B) experiment 2:129P2 and 129X1 mice.

Significant trial and trial x strain interactions effects were found for the latency until the first board entry (trial: F_19, 304 = _11.917, P<0.001; strain x trial: F_19,304 = _5.334, P<0.001), the total time spent on the board (trial: F_19, 303.2 = _13.531, P<0.001; strain x trial: F_19,303.2 = _1.694, P<0.05) and the total number of board entries (trial: F_19,303.2 = _5.933, P<0.001; strain x trial: F_19,303.2 = _2.549, P<0.001). Both strains showed an increase in the latency until the first board entry across the experimental period (fig. 1A), in addition the total time spent on the board and the number of board entries (fig. 2A) decreased across the experimental time period, thereby showing increased avoidance behaviour of the unprotected area.

#### Experiment 2: 129X1 vs. 129P2

Significant strain (F_1,16 = _5.21, P<0.05) and trial effects (F_19,302.2 = _20.38, P<0.01) were found for the latency until the first board entry. Both strains showed an increase in the latency until the first board entry across the experimental period (fig. 1B). The time spent on the board (fig. 2B) only showed a significant trial (F_19,302.2 = _10.98, P<0.01) effect and both strains showed a decrease in time spent on the board across the experimental period. Significant strain (F_1,16 = _11.62, P<0.05), trial (F_19,302.5 = _15.39, P<0.01) and strain x trial interaction (F_19,302.5 = _3.35, P<0.01) effects were found for the number of board entries. In both strains, the number of board entries decreased across the experimental period. In general, both strains thus showed increased avoidance behaviour over time.

### Risk Assessment

#### Experiment 1:129S2Pas vs. 129S2Hsd

Significant trial effects were found for the total number of stretched attends (F_19,200 = _32.007, P<0.001) and the latency until the first stretched attend (F_19,204 = _4.368, P<0.001). Both strains showed an increase in latency until the first stretched attend (129S2Pas: 10.2±5.8 in trial1, 212.6±122.2 in trial 20; 129S2Hsd: 7.3±3.2 in trial 1, 197.7±89.5 in trial 20) and a general decrease in stretched attend postures (129S2Pas: 7±12 in trial1, 4±0.5 in trial 20; 129S2Hsd: 2±17.5 in trial 1, 3±1 in trial 20) across the experimental period.

#### Experiment 2:129X1 vs. 129P2

Significant trial effects were found for the total number of stretched attends (F_19,302.7 = _14.41, P<0.01) and the latency until the first stretched attend (F_19,302.7 = _10.778, P<0.001). Both strains showed an increase in latency until the first stretched attend (129X1: 52.3±33.9 in trial1, 279.1±19.0 in trial 20; 129P2: 42.3±36.8 in trial 1, 300±0 in trial 20) and a general decrease in stretched attends postures (129X1: 6±10 in trial1, 0±1 in trial 20; 129P2: 7±12 in trial 1, 0±0 in trial 20) across the experimental period.

### Locomotor Activity

#### Experiment 1: 129S2Pas vs. 129S2Hsd

**Figure 3:**
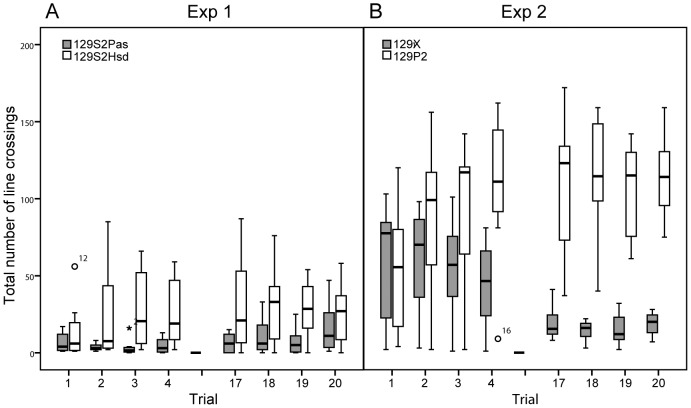
Total number of line crosses (median ± IQR) on the first day and last day of testing (trials 1–4 and 17–20). (3A) Experiment 1: 129S2Pas and129S2 mice, (3B) experiment 2: 129X1 and 129P2 mice.

Significant strain (F_1,23.6 = _4.363, P<0.05) and strain x trial effects (F_19,303.2 = _2.701, P<0.001) were found for the total number of line crossings (fig. 3A). 129S2Pas mice initially showed less line crossings compared to 129S2Hsd mice. Further, significant strain (F_1,19.4 = _11.896, P<0.001), trial (F_19,207.8 = _4.445, P<0.001) and strain x trial effects (F_19,207.8 = _3.587, P<0.001) were found for the latency until the first line crossing, which decreased across the experimental period in both strains. Locomotor activity was further analysed by immobility events. Significant strain and trial effects were found for the total time spent immobile (strain: F_1,21.8 = _12.163, P<0.001; trial: F_19,215.1 = _5.814, P<0.001) and the latency until the first immobility (strain: F_1,21.8 = _7.222, P<0.001; trial: F_19,215.1 = _10.278, P<0.001). Both strains showed increased immobility across the experimental period (129S2Pas: 3.6±1.6% in trial 1, 39.3±6.7% in trial 20; 129S2Hsd: 0.1±0.1% in trial 1, 17.9±5.9% in trial 20), whereas 129S2Pas mice overall spent more immobile than 129S2Hsd mice (table S1).

#### Experiment 2: 129X1 vs. 129P2

Significant strain, trial and strain x trial effects were found for the total number of line crossings (fig. 3B, strain: F_1,16 = _37.80, P<0.001; trial: F_19,302.1 = _2.60, P<0.001; strain x trial: F_19,302.0 = _7.67, P<0.001) and the latency until the first line crossing (strain: F_1,16 = _25.58, P<0.001; trial: F_19,302.0 = _6.56, P<0.001; strain x trial: F_19,302.0 = _5.02, P<0.001). Whereas 129X1 mice showed a decrease in line crossings, 129P2 mice showed a general increase in line crossings across the experimental period.

Significant effects were further found for immobility duration (strain: F_1,16 = _40.76, P<0.001; trial: F_19,302.0 = _19.12, P<0.001; strain x trial: F_19_,_302.0 = _10.47, P<0.001) and latency until the first immobility event (strain: F_1,16 = _12.88, P<0.001; trial: F_19,302.0 = _19.52, P<0.001; strain x trial: F_19,302.0 = _2.10, P<0.01). Both strains showed increased immobility duration over time (129X1: 0±0% in trial 1, 56.5±6.3% in trial 20; 129P2: 0±0% in trial 1, 7.9±3.7% in trial 20), however 129X1 mice were more immobile than 129P2 mice (table S1).

### General Exploration

#### Experiment 1: 129S2Pas vs. 129S2Hsd

Significant strain effects were found for the total number of rearings (F_1,20.8 = _5.900, P<0.05) and latency until the first rearing in the box (F_1,20.8 = _11.762, P<0.001). 129S2Hsd mice showed more rearings than 129S2Pas mice. More specifically, there was an increase in rearings over time in 129S2Hsd mice (trial 1: 3±1.5, trial 20: 8±5.5) and the amount of rearings in 129S2Pas mice stayed at the same level over time (trial 1: 3±2, trial 20: 1±1.5). The latency until the first rearing increased during the experimental period (129S2Pas: 98.6±33.8 in trial1, 210.0±60.7 in trial 20; 129 S2Hsd: 128.3±13.9 in trial 1, 184.2±16.5 in trial 20), indicated by a general trial effect (F_19,304 = _2.396, P<0.001). No significant effects were found for the number of rearings on the board or latency until first rearing on the board, since both strains hardly displayed this behaviour. Significant trial effects were found for the number of hole explorations and (F_19,304 = _15.934, P<0.001) and latency until the first hole exploration (F_19,304 = _6.746, P<0.001). Both strains showed a general decrease in hole exploration (129S2Pas: 16±9.5 in trial1, 4±2 in trial 20; 129 S2Hsd: 9±12 in trial 1, 2±1 in trial 20) across the experimental period.

#### Experiment 2: 129X1 vs. 129P2

Significant strain x trial interaction (F_19,302.0 = _5.89, P<0.01) effects were found for the number of rearings in the box. Whereas the number of rearings in 129X1 mice remained unchanged (trial 1: 3±11, trial 20: 3±5), 129P2 mice showed an increased number of rearings across the experimental period (trial 1: 0±1, trial 20: 12±9). Significant trial (F_19,302.0 = _2.164, P<0.01) and strain x trial interaction (F_19,302.0 = _2.335, P<0.01) effects were found for the latency until the first rearing in the box, both strains showed an increase in latency across the experimental period (129X1: 169.0±35.3 in trial1, 137.3±26.2 in trial 20; 129P2: 242.8 169.0±32.0 in trial 1, 120.0±31.8 in trial 20). Both 129P2 and 129X1 mice hardly displayed any rearings on the board, so no significant effects on numbers or latency regarding this behaviour were found. Significant trial effects for the number of hole explorations (F_19,302.0 = _16.37, P<0.001) and the latency until the first hole exploration (F_19,302.0 = _23.15, P<0.001) were found. Both strains showed a clear decrease in number of hole explorations (129X1: 13.5±26 in trial1, 0±1 in trial 20; 129P2: 15.5±9 in trial 1, 1.5±4 in trial 20) and increased latency to explore holes across the experimental period (table S1).

### Directed Exploration

#### Experiment 1: 129S2Pas vs. 129S2Hsd

No significant effects were found on the number of hole visits, since both strains hardly visited the holes. Nevertheless, a significant trial effect (F_19,304 = _2.303, P<0.01) was found for latency until the first hole visit, as both strains showed a small decrease in latency across the experimental period (table S1).

#### Experiment 2: 129X1 vs. 129P2

A significant strain effect (F_19,318.0 = _5.58, P<0.05) was found for the number of hole visits, although no significant effects were found for the latency until the first hole visit and both strains hardly visited the holes (table S1).

### Food Intake Inhibition

#### Experiment 1: 129S2Pas vs. 129S2Hsd

Significant strain effects (F_1,16 = _5.533, P<0.001) were found for the latency until the first exploration of the unfamiliar food, as 129S2Hsd mice showed a lower latency (trial 1: 256.7±22.1, trial 20: 273.0±27.0) than 129S2Pas mice (trial 1: 300.0±0, trial 20: 300.0±0). Strain (F_1,17 = _9.371, P<0.01), trial (F_19,199.6 = _2.447, P<0.01) and strain x trial effects (F_19;199.6 = _2.181, P<0.01) were found for the latency until the first exploration of the familiar food. Only the 129S2Hsd strain showed a decrease in latency to explore the familiar food (129S2Hsd: 205.6±34.6 in trial 1, 155.0±36.4 in trial 20; 129S2Pas: 276.6±23.2 in trial 1, 244.4±27.3 in trial 20). 129S2Hsd mice showed a lower latency compared to 129S2Pas.

#### Experiment 2: 129X1 vs. 129P2

A significant trial (F_19,302.0 = _3.79, P<0.01) and strain x trial interaction (F_19,302.0 = _3.67, P<0.01) effect was found for the latency until the first exploration of the familiar food. 129P2 mice showed a clear decrease in latency across the experimental time period (trial 1: 266.5±23.5, trial 20: 152.5±47.0), whereas this was not observed in 129X1 mice (trial 1: 186.6±44.4, trial 20: 253.9±30.2). This was also found for the latency until the first exploration of the unfamiliar food (trial: F_19,302.0 = _1.92, P<0.05; strain: F_1,16 = _5.062, P<0.05). 129P2 mice showed a decrease in latency across the experimental period in contrast to 129X1 mice that did not show this decrease (129P2: 193.6±37.1 in trial 1, 60.9±35.0 in trial 20; 129 X1: 183.7±44.2 in trial 1, 181.4±35.7 in trial20).

### Arousal/De-Arousal

#### Experiment 1: 129S2Pas vs. 129S2Hsd

Significant trial and strain x trial effects were found for the total time spent grooming (trial: F_19,304 = _3.173, P<0.001; strain x trial: F_19,304 = _2.386, P<0.001), the latency until the first self-grooming event (trial: F_19,304 = _3.141, P<0.001; strain x trial: F_19,304 = _1.908, P<0.05) and the total number of self-grooming bouts (trial: F_19,304 = _2.861, P<0.001; strain x trial: F_19,304 = _1.784, P<0.05). Both strain showed an increase in grooming behaviour across the experimental period (129S2Pas: 0.0±0% in trial 1, 0.3±0.2% in trial 20; 129S2Hsd: 0.0±0% in trial 1, 2.1±0.4% in trial 20).

No significant effects were found for the number of defecations.

#### Experiment 2: 129X1 vs. 129P2

Significant trial, strain and strain x trial interaction effects were found for the total time spent grooming (trial: F_19,302.0 = _3.58, P<0.01; strain: F_1,16 = _11.76, P<0.05; strain x trial: F_19,302.0 = _2.17, P<0.05), the latency until the first grooming event (trial: F_19,302.0 = _5.23, P<0.01; strain: F_1,16 = _27.97, P<0.01; strain x trial: F_19,302.0 = _2.45, P<0.01) and the number of grooming events (strain: F_1,16 = _19.04, P<0.01; trial: F_19,302.0 = _3.29, P<0.01). Both strains showed an increase in grooming duration (129X1: 0.2±0.2% in trial 1, 5.0±2.8% in trial 20; 129P2: 0.4±0.2% in trial 1, 0.6±0.3% in trial 20) and grooming events, although this was clearer for 129X1 mice (table S1).

Significant trial (F_19,302.0_ = 3.06, P<0.001) and strain x trial interaction (F_19,302.0_ = 1.74, P<0.05) effects were found for the number of defecations (table S1), whereas only 129X1 mice showed a decrease in number of produced boli during the experimental period (trial 1: 6±3, trial 20: 3±2).

### Corticosterone

Corticosterone data is represented in table 1.

**Table 1:** Plasma corticosterone (CORT) levels before and after testing in both experiments.

strain	statistics	pCORT	
		Before testing	After testing
**Experiment 1**			
129S2Pas	ns	371.06±123.85	245.18±60.95
129S2Hsd	ns	324.86±92.99	144.41±29.46
**Experiment 2**			
129X1	ns	79.74±28.19	137.65±48.67
129P2	ns	210.06±74.27	103.51±36.60

Data is represented in nmol/liter (± SEM) ns = non significant effect.

#### Experiment 1: 129S2Pas vs. 129S2Hsd

No significant differences were found between basal/non-basal CORT values or between the two strains.

#### Experiment 2: 129X1 vs. 129P2

No significant differences were found between basal/non-basal CORT values within both strains nor were there significant strain differences. Non-basal values of 129P2 mice showed a trend to be higher than those of basal levels, although this difference did not reach significance (t = −2.602, p = 0.032, corrected p<0.0167).

## Discussion

Like previously reported for 129P3/J mice [20], [24], [32], the four substrains of the 129 family tested in the present study showed a lack of habituation of avoidance behaviour towards an initially novel area over time. Thus the 129 mouse strain seems to be characterized by a distinct profile that implies difficulties coping with environmental changes, although distinct substrain differences were found at the behavioural level as well. Notably, habituation reflects the adaptive process of integrating emotional and cognitive processes in order to enable an organism to adequately respond to changes in the environment, (e.g. waning of an initial anxiety response after repeated exposure to the same stimulus [23], [33]). A slow or impaired habituation can then be understood to indicate an inability to adapt and might therefore endanger an animals' welfare if it is exposed to (even mild) environmental challenges [24]. Based on this hypothesis it has to be concluded that guidelines regarding husbandry and experimental procedures should account for strain- or even substrain-specific adaptive capacities in laboratory mice.

In previous studies in which an identical test set-up and experimental procedure was used, C57BL6N male mice revealed a low level of avoidance behaviour during the initial exposure as well as over time [13], [31], while both DBA2 [13] and BALB/c [24] mice displayed a high initial avoidance followed by a decrease in avoidance behaviour over time resulting in a stable baseline-level around the third day of testing. In contrast, all 129 substrains tested in previous [20], [24], [32] and the present study fail to reveal a decrease in avoidance behaviour and even show an increase of this behaviour over time (fig. 1 and 2).

For a correct interpretation of such a behavioural phenomenon it is important to exclude potential confounding factors of the readout parameters. A lack of exploration of a distinct area for example might as well be the result of a low level of overall activity. The persisting level of high avoidance behaviour in 129 substrains therefore might have been a secondary effect to changes in other behavioural domains. Thus, in addition to different parameters indicating avoidance behaviour, we simultaneously investigated general exploratory and locomotor activity and found different behavioural profiles in the 129 mouse substrains. At first animals of the 129P2 substrain revealed only minor changes in locomotor activity over time (fig. 3, table S1), which is comparable to the previously tested 129P3 mice [24], [32]. Additionally an increase in general exploratory activity (number of rearings) over time was found in this substrain (table S1), indicating that overall activity levels did not cause the increase in avoidance behaviour over time. Rather, this behavioural profile indicates that animals of the 129P2 substrain indeed are likely to be limited in their ability to adapt to novelty.

In contrast, 129S2 (129S2Hsd and 129S2Pas) mice were generally more inactive in comparison with the other 129 substrains. Especially 129S2Pas mice were immobile for about 50% of the total testing time. Further, they showed high latencies of the first line crossing and a low total number of line crossings already after the first couple of trials (fig. 3, table S1). In addition the rearing activity in this substrain was very low (table S1). Both the 129S2Hsd and the 129S2Pas substrain revealed, in accordance with the findings of others [30], , low levels of locomotor and exploratory activity. A final interpretation of habituation profiles in these substrains thus remains difficult since either high avoidance behaviour may be caused by a low general activity, or this low general activity might be caused by high anxiety via behavioural inhibition [37].

Conversely, the 129X1 substrain showed a more gradual increase in immobility and decrease in locomotor activity over time than the 129S2 substrains. Those animals initially displayed by equal amounts of line crossings as 129P2 mice, but locomotor activity decreased over time (fig. 3, table S1) as found before by Tang et al. [27]. In consistency with findings on locomotor activity, the number of rearings decreased over time (table S1). This profile may suggest that the animals' during initial trials did gather sufficient information to conclude that there was no further need to explore the testing environment. However, the general decrease in activity makes it difficult to assess if the increase of avoidance behaviour seen in 129X1 mice can be considered adaptive (i.e. an adequate response to sufficient information gathering) or non-adaptive (i.e. an inability to overcome novelty-induced avoidance).

Based on the fact that habituation is a cognitive process [23], [33], [38] one may argue that a lack of habituation can be based on primary cognitive deficits. Indeed some 129 substrains have been described to be impaired in novel object learning when compared to other strains [36], [39], [40]. However, in a previous experiment it was shown that 129P3 mice were able to discriminate between a novel and a familiar object in a 1-trial object recognition paradigm [24]. A comparable indication of 1-trial recognition abilities is integrated in the test procedure used in the present study by investigating the level of recognition of a familiar food object in comparison with an unfamiliar food object in the first trial [31]. However, in none of the substrains tested here a significant discrimination between the two food objects could be observed during the first trial, which might be the result of the relatively high initial behavioural inhibition in all animals, resulting in exploration of both the familiar and the unfamiliar food only late or not at all (table S1). However, further investigation on cognitive abilities of the 129P2 strain (that showed a high discriminative learning over time in the present experiment) in the cognitive version of the modified hole board [41] indicates that at least spatial cognition is not impaired in this substrain, indicating that the increase in avoidance behaviour over time as seen here is probably not caused by cognitive dysfunctions.

The present study revealed similarities, but also considerable substrain specific differences in behaviour, comparable to other studies [26]–[30]. It is important to consider that the behavioural phenotype of animals is not only determined by genetic background but also by environment and gene-environment interactions [16], [17]. Notably, 129S2Hsd and 129S2Pas mice tested in the present study derive from the same 129S2/Sv strain, but are being kept as separate breeding colonies at different breeders. Thus, differences in behaviour as found here might be due to different environmental conditions at the breeding facilities as well as genetic drift [42].

However, we also found that the behavioural profile of 129P2 mice is comparable to that of the 129P3 strain tested in earlier studies [20], [24], [32]. Although these two strains are closely related [26] 129P3 and 129P2 mice are derived from different breeders as well. The fact that two genetically similar substrains (129S2HSd and 129S2Pas) can considerably differ, while two genetically different substrains (129P3 and 129P2) show similar profiles may be the result of innumerable environmental and genetic factors which are difficult to pinpoint [43].

In line of this reasoning and based on our substrain comparison, we conclude, however, that the genetic background of the 129 substrains is likely to cause an increased vulnerability for a quite limited ability to adapt to novel environments.

### Welfare implications

Processes of adaptation are considered of potential relevance for our understanding of animal welfare: The concept of allostasis for example [7] states that an animal's welfare is not impaired as long as animals are able to adapt to (changing) environmental challenges such as exposure to novelty. Thus a high anxiety characteristic might not necessarily be detrimental to an animal's state of welfare as long as an individual is able to adapt, resulting in a decrease in anxiety towards a specific challenge over time [4], [6], [8]. Given that the process of selection and inbreeding in laboratory mice has resulted in the (unintentional) co-selection of emotional traits (see for example [8], [11], [13], [14], [44], [45]), we hypothesize that standard housing and treatment protocols for mice as a species may be insufficient to safeguard the welfare of different mouse (sub)strains equally. Compared to other strains, different 129 substrains reveal a reduced ability to adapt to exposure to novelty. In addition others have found that several 129 substrains (including 129P3 and 129X1) also show impaired fear extinction [46], [47]. Together with our results, this suggests that several 129 substrains have difficulties to cope with mild and more severe negative events. Although the question whether the welfare state of these mice might be generally compromised under standard laboratory housing conditions remains to be investigated, it is to be expected that environmental changes (such as transport between facilities or rooms, changes of light-regime, cage cleaning, or enrichment replacement) might be of higher impact on 129 substrains than in other strains. It might therefore be useful to consider guidelines and protocols that take into account the adaptive capabilities of mice at the strain- or even substrain-level.

## Supporting Information

Table S1 **Overview of the behavioural data in both experiments; data from the first (trial 1) and last (trial 20) trial are shown.** Continuous data are represented as mean ± SEM and discrete data as median ± IQR. Mixed model analysis was used, the best fitting model was used per behavioural parameter (1 m = fixed model (fixed intercept/fixed slope), 2 m = random intercept (random intercept/fixed slope), 3 m = random intercept and slope (random intercept/random slope)). P<0.05 was considered significant. nr: number, s: seconds, T: trial, S: strain, S*T: strain x trial interaction and ns: non significant.
(EPS)

## References

[1] Brambell FWR (1965) Report of the technical committee to enquire into the welfare of animals kept under intensive livestock husbandry systems.

[2] Boissy A, Manteuffel G, Jensen MB, Moe RO, Spruijt B, et al (2007) Assessment of positive emotions in animals to improve their welfare. Physiol Behav 92(3): 375–397. 17428510 10.1016/j.physbeh.2007.02.003

[3] Yeates JW, Main DCJ (2008) Assessment of positive welfare: A review. Vet J 175 (3) 293–300. 17613265 10.1016/j.tvjl.2007.05.009

[4] Ohl F, van der Staay FJ (2012) Animal welfare: At the interface between science and society. Vet J 192 (1) 13–19. 21703888 10.1016/j.tvjl.2011.05.019

[5] Bracke MBM, Hopster H (2006) Assessing the importance of natural behavior for animal welfare. J Agric Environ Ethics 19 (1) 77–89.

[6] Salomons AR, Arndt SS, Ohl F (2009) Anxiety in relation to animal environment and welfare. Scand J Lab Anim Sci 36 (1) 37–45.

[7] Korte SM, Olivier B, Koolhaas JM (2007) A new animal welfare concept based on allostasis. Physiol Behav 92 (3) 422–428. 17174361 10.1016/j.physbeh.2006.10.018

[8] Ohl F, Arndt SS, van der Staay FJ (2008) Pathological anxiety in animals. Vet J 175 (1) 18–26. 17321766 10.1016/j.tvjl.2006.12.013

[9] Knierim U, Winckler C (2009) On-farm welfare assessment in cattle: Validity, reliability and feasibility issues and future perspectives with special regard to the welfare quality® approach. Anim Welf 18 (4) 451–458.

[10] Webster AJF (1994) Animal welfare: A cool eye towards eden. Londen, UK: Blackwell Science ltd.

[11] Jensen P (2010) Domestication, selection, behaviour and welfare of animals - genetic mechanisms for rapid responses. Anim Welf 19 (SUPPL. 1) 7–9.

[12] Belzung C, Griebel G (2001) Measuring normal and pathological anxiety-like behaviour in mice: A review. Behav Brain Res 125 (1–2) 141–149. 11682105 10.1016/s0166-4328(01)00291-1

[13] Ohl F, Roedel A, Binder E, Holsboer F (2003) Impact of high and low anxiety on cognitive performance in a modified hole board test in C57BL/6 and DBA/2 mice. Eur J Neurosci 17: 128–136. 12534976 10.1046/j.1460-9568.2003.02436.x

[14] Laarakker MC, Ohl F, Van Lith HA (2008) Chromosomal assignment of quantitative trait loci influencing modified hole board behavior in laboratory mice using consomic strains, with special reference to anxiety-related behavior and mouse chromosome 19. Behav Genet 38 (2) 159–184. 18175213 10.1007/s10519-007-9188-6

[15] Clément Y, Guisquet AML, Venault P, Chapouthier G, Belzung C (2009) Pharmacological alterations of anxious behaviour in mice depending on both strain and the behavioural situation. PLoS One 4 (11) e7745. 19907641 10.1371/journal.pone.0007745 PMC2770638

[16] Crabbe JC, Wahlsten D, Dudek BC (1999) Genetics of mouse behavior: Interactions with laboratory environment. Science 284 (5420) 1670–1672. 10356397 10.1126/science.284.5420.1670

[17] van der Staay FJ, Arndt SS, Nordquist RE (2010) The standardization-generalization dilemma: A way out. Genes Brain Behav 9 (8) 849–855. 20662940 10.1111/j.1601-183X.2010.00628.x

[18] Abramov U, Puussaar T, Raud S, Kurrikoff K, Vasar E (2008) Behavioural differences between C57BL/6 and 129S6/SvEv strains are reinforced by environmental enrichment. Neurosci Lett 443 (3) 223–227. 18687379 10.1016/j.neulet.2008.07.075

[19] Pothion S, Bizot J, Trovero F, Belzung C (2004) Strain differences in sucrose preference and in the consequences of unpredictable chronic mild stress. Behav Brain Res 155 (1) 135–146. 15325787 10.1016/j.bbr.2004.04.008

[20] Salomons AR, Kortleve T, Reinders NR, Kirchhoff S, Arndt SS, et al (2010) Susceptibility of a potential animal model for pathological anxiety to chronic mild stress. Behav Brain Res 209 (2) 241–248. 20138918 10.1016/j.bbr.2010.01.050

[21] Ohl F, Roedel A, Storch C, Holsboer F, Landgraf R (2002) Cognitive performance in rats differing in their inborn anxiety. Behav Neurosci 116 (3) 464–471. 12049327 10.1037//0735-7044.116.3.464

[22] Livesey PJ (1986) Learning and emotion: A biological synthesis. Hillsdale, New Jersey: Lawrence Erlbaum Associates, publishers.

[23] Eisenstein EM, Eisenstein D (2006) A behavioral homeostasis theory of habituation and sensitization: II. further developments and predictions. Rev Neurosci 17 (5) 533–557. 17180878 10.1515/revneuro.2006.17.5.533

[24] Salomons AR, van Luijk JAKR, Reinders NR, Kirchhoff S, Arndt SS, et al (2010) Identifying emotional adaptation: Behavioural habituation to novelty and immediate early gene expression in two inbred mouse strains. Genes Brain Behav 9(1): 1–10. 19751395 10.1111/j.1601-183X.2009.00527.x

[25] National Research Council (2010) Guide for the care and use of laboratory animals. Washington, DC: National Academies Press.

[26] Simpson EM, Linder CC, Sargent EE, Davidsson MT, Mobraaten LE, et al (1997) Genetic variation among 129 substrains and its importance for targeted mutagenesis in mice. Nat Genet 16. 10.1038/ng0597-19 9140391

[27] Tang X, Sanford LD (2005) Home cage activity and activity-based measures of anxiety in 129P3/J, 129X1/SvJ and C57BL/6J mice. Physiol Behav 84 (1) 105–115. 15642613 10.1016/j.physbeh.2004.10.017

[28] Bothe GWM, Bolivar VJ, Vedder MJ, Geistfeld JG (2005) Behavioral differences among fourteen inbred mouse strains commonly used as disease models. Comp Med 55 (4) 326–334. 16158908

[29] Bothe GWM, Bolivar VJ, Vedder MJ, Geistfeld JG (2004) Genetic and behavioral differences among five inbred mouse strains commonly used in the production of transgenic and knockout mice. Genes Brain Behav 3 (3) 149–157. 15140010 10.1111/j.1601-183x.2004.00064.x

[30] Cook MN, Bolivar VJ, McFadyen MP, Flaherty L (2002) Behavioral differences among 129 substrains: Implications for knockout and transgenic mice. Behav Neurosci 116 (4) 600–611. 12148927

[31] Ohl F, Sillaber I, Binder E, Keck ME, Holsboer F (2001) Differential analysis of behavior and diazepam-induced alterations in C57BL/6N and BALB/c mice using the modified hole board test. J Psychiatr Res 35: 147–154. 11461710 10.1016/s0022-3956(01)00017-6

[32] Salomons AR, Bronkers G, Kirchhoff S, Arndt SS, Ohl F (2010) Behavioural habituation to novelty and brain area specific immediate early gene expression in female mice of two inbred strains. Behav Brain Res 215 (1) 95–101. 20615435 10.1016/j.bbr.2010.06.035

[33] Bolivar VJ (2009) Intrasession and intersession habituation in mice: From inbred strain variability to linkage analysis. Neurobiol Learn Mem 92 (2) 206–214. 19496240 10.1016/j.nlm.2009.02.002 PMC2772117

[34] Pratte M, Jamon M (2009) Detection of social approach in inbred mice. Behav Brain Res 203 (1) 54–64. 19379777 10.1016/j.bbr.2009.04.011

[35] de Visser L, van den Bos R, Kuurman WW, Kas MJH, Spruijt BM (2006) Novel approach to the behavioural characterization of inbred mice: Automated home cage observations. Genes Brain Behav 5 (6) 458–466. 16923150 10.1111/j.1601-183X.2005.00181.x

[36] Sik A, van Nieuwehuyzen P, Prickaerts J, Blokland A (2003) Performance of different mouse strains in an object recognition task. Behav Brain Res 147 (1–2) 49–54. 14659569 10.1016/s0166-4328(03)00117-7

[37] Gray JA (1982) The neuropsychology of anxiety. Oxford and New York: Oxford University Press New York, Clarendon Press Oxford.

[38] O'Keefe J (1999) Do hippocampal pyramidal cells signal non-spatial as well as spatial information? Hippocampus 9 (4) 352–364. 2-1. 10495018 10.1002/(SICI)1098-1063(1999)9:4<352::AID-HIPO3>3.0.CO;2-1

[39] Montkowski A, Poettig M, Mederer A, Holsboer F (1997) Behavioural performance in three substrains of mouse strain 129. Brain Res 762 (1–2) 12–18. 9262153 10.1016/s0006-8993(97)00370-3

[40] Kim D, Chae S, Lee J, Yang H, Shin HS (2005) Variations in the behaviors to novel objects among five inbred strains of mice. Genes Brain Behav 4 (5) 302–306. 16011576 10.1111/j.1601-183X.2005.00133.x

[41] Salomons AR, Arndt SS, Ohl F (2012) Impact of anxiety-profiles on cognitive performance in BALB/c and 129P2 mice. Cogn Affect and Behav Neurosci in press. 10.3758/s13415-012-0109-7 PMC3505495 22760949

[42] Casellas J (2011) Inbred mouse strains and genetic stability: A review. Anim 5 (1) 1–7. 10.1017/S1751731110001667 22440695

[43] Chesler EJ, Wilson SG, Lariviere WR, Rodriguez-Zas SL, Mogil JS (2002) Identification and ranking of genetic and laboratory environment factors influencing a behavioral trait, thermal nociception, via computational analysis of a large data archive. Neurosci Biobehav Rev 26 (8) 907–923. 12667496 10.1016/s0149-7634(02)00103-3

[44] Belzung C, Berton F (1997) Further pharmacological validation of the BALB/c neophobia in the free exploratory paradigm as an animal model of trait anxiety. Behav Pharmacol 8 (6–7) 541–548. 9832968 10.1097/00008877-199711000-00012

[45] Clément Y, Calatayud F, Belzung C (2002) Genetic basis of anxiety-like behaviour: A critical review. Brain Res Bull 57 (1) 57–71. 11827738 10.1016/s0361-9230(01)00637-2

[46] Camp M, Norcross M, Whittle N, Feyder M, D'Hanis W, et al (2009) Impaired pavlovian fear extinction is a common phenotype across genetic lineages of the 129 inbred mouse strain. Genes Brain Behav 8 (8) 744–752. 19674120 10.1111/j.1601-183X.2009.00519.x PMC2783364

[47] Hefner K, Whittle N, Juhasz J, Norcross M, Karlsson R, et al (2008) Impaired fear extinction learning and cortico-amygdala circuit abnormalities in a common genetic mouse strain. J Neurosci 28 (32) 8074–8085. 18685032 10.1523/JNEUROSCI.4904-07.2008 PMC2547848

